# Editorial: Biotyping in Psychiatry

**DOI:** 10.3389/fpsyt.2022.844206

**Published:** 2022-02-02

**Authors:** Tae Young Lee, Hang Joon Jo, Shinsuke Koike, Andrea Raballo

**Affiliations:** ^1^Department of Psychiatry, Pusan National University Yangsan Hospital, Yangsan, South Korea; ^2^Research Institute for Convergence of Biomedical Science and Technology, Pusan National University Yangsan Hospital, Yangsan, South Korea; ^3^Department of Biomedical Engineering, Hanyang University Graduate School of Biomedical Science and Engineering, Seoul, South Korea; ^4^Department of Physiology, Hanyang University College of Medicine, Seoul, South Korea; ^5^Department of Neuropsychiatry, Graduate School of Medicine, The University of Tokyo, Tokyo, Japan; ^6^Center for Evolutionary Cognitive Sciences (ECS), Graduate School of Art and Sciences, The University of Tokyo, Tokyo, Japan; ^7^Division of Psychiatry, Department of Medicine, University of Perugia, Perugia, Italy; ^8^Center for Translational, Phenomenological and Developmental Psychopathology, Perugia University Hospital, Perugia, Italy

**Keywords:** biotyping, biomarker, RDoC, psychiatry, NIMH

Since Wilhelm Griesinger's famous statement that “all mental illnesses are cerebral illnesses” (aka brain mythology), there have been recursive calls for a global revision of psychiatric classifications to better accommodate for mental disorders as disorders of the brain (1). However, the cleavage between current diagnostic systems for mental disorders (which rely upon presenting signs and symptoms) and contemporary neuroscience has not been bridged, to the point that the Research Domain Criteria (RDoC) paradigm was launched as an alternative approach to optimize the identification of relevant neurobiological and behavioral systems involved in the pathogenesis of mental disorders (2). The basic inspiration of the RDoC was to reclassify mental diseases based on biological markers ranging from genes to circuits, physiology, behavior. Specifically, the central heuristic architecture of the RDoC is the deconstruction of human behavior and brain function into neuropsychological “domains” (i.e., “negative valence systems,” “positive valence systems,” “cognitive systems,” “social processes,” “arousal and regulatory systems,” and “sensorimotor”) and related subcomponents, thereby facilitating the identification of multilevel neurobiological substrates.

This move was essentially motivated by the empirical observation that polythetic diagnostic systems for mental disorders, such as the DSM and ICD, are taxed by high degrees of inter-class overlaps, comorbidity and heterogeneity, as well as diverse disease course and response to treatment within the same diagnosis (3). However, although the RDoC initiative was launched by the National Institute of Mental Health more than a decade ago, the gap between traditional research based on syndromic classification and RDoC-based investigation remains monumental and largely unaddressed (4). An obvious, pragmatic strategy to reduce such gap (and finally actualize Griesinger's hope) is to progressively move toward a hybrid system systematically enriching the biological fingerprints of present diagnostic categories, given that a fully biomarker-driven diagnostic system is still a rather distant and futuristic goal. Such a process will necessitate the iterative refinement of interim diagnostic systems and the establishment of standardized methodologies to maximize generalizability. For example, adopting a dimensional, trans-diagnostic perspective to reclassify symptoms could help the understanding of the pathophysiology of psychiatric illnesses in terms of onset, syndromic aggregation of signs and symptoms, and later outcomes. This might also inspire more precise treatment targets or preventive interventions. Therefore, this special Research Topic addresses promising new avenues centered around biotyping in psychiatry. The nine collected studies (2 systematic reviews, 1 mini-review, and 6 original investigations) specifically focus on biotyping in order to redefine or reclassify existing mental diseases or to identify biomarkers necessary for such biotyping.

As per the two systematic reviews, first Fatih et al. addressed long-term intracortical inhibition (LICI) as a biomarker in neuropsychiatric disorders. They reviewed 113 articles on psychiatric disorders as well as neurologic disorders. The results indicate that although LICI may have utility as a biomarker of GABA_B_ functioning, many studies present heterogenous methodology and inconsistent findings, thereby requiring a more substantial effort to increase shared standards in the field. Second, Miranda et al. conducted a systematic review of functional magnetic resonance imaging from the perspective of unsupervised machine learning applications for disease subtyping. However, the results for all explored diseases are inconsistent, indicating the need for concerted, multisite data collection in order to measure the generalizability of results.

The mini-review by Sugiyama et al. addresses the electrophysiological index for sensory processing dysfunctions in psychiatric disorders on the basis of findings of the auditory steady-state response (ASSR). They propose that ASSR amplitude, phase, and resetting responses are sensitive indices for investigating sensory processing dysfunction in psychiatric disorders.

As per the six empirical contributions presented in this topic, two focus on hippocampal subfield studies. Sasabayashi et al. conducted a hippocampal subfield volumetry across illness stages. They suggested that the reduced hippocampal subfield volumes may represent a common biotype associated with psychosis vulnerability. On the other hand, Tai et al. investigated the pathophysiology which protects against progressive hippocampal atrophy by altering neuronal plasticity or inducing neurogenesis. Egger et al. conducted a functional transcranial Doppler study of cerebral blood flow velocity patterns in patients with schizophrenia. The results support the view that schizophrenia, particularly symptom load and thus severity, influences performance in neurocognitive tasks whilst being related to distinct brain hemodynamic patterns. Takahashi et al. conducted an eye movements investigation as a non-invasive potential biomarker for the diagnosis of major depressive disorder. Free-viewing test, Lissajous trajectories of the smooth pursuit eye movement test, and fixation stability test were adopted. They suggested that the detailed parameters of eye movements can assist in differentiating depressive patients from healthy comparisons. Koshiyama et al. investigated an identification of the neural sources and their dynamic interactions using resting-state electroencephalography. This study provides evidence that abnormal resting-state electroencephalography oscillations are driven by patterns of hyper-connectivity across multiple frequency bands and a distributed network of the frontal, temporal and occipital brain regions that are involved in visual and auditory information processing in schizophrenia patients. Dong et al. investigated the prefrontal hemodynamics of patients with major depressive disorders using a head-mounted functional near-infrared spectroscopy.

Taken together, these articles explored state-of-the-art approaches to identify biomarkers or biotypes using a variety of methods, including long-term intracortical inhibition, resting-state or task-based functional connectivity, functional transcranial Doppler. However, to accelerate progress and minimize the risk of inconsistent results and generalizability problems, the next wave of biotyping research should establish standardized methods and adopt transdiagnostic approaches with a sufficient sample size.

## Author Contributions

TYL and AR wrote the manuscript. All authors provided important intellectual contributions, read, and approved the final version.

## Funding

This work was supported by the National Research Foundation of Korea (NRF) grant funded by the Korea government (MSIT) (No. 2021R1A2C1006718).

## Conflict of Interest

The authors declare that the research was conducted in the absence of any commercial or financial relationships that could be construed as a potential conflict of interest.

## Publisher's Note

All claims expressed in this article are solely those of the authors and do not necessarily represent those of their affiliated organizations, or those of the publisher, the editors and the reviewers. Any product that may be evaluated in this article, or claim that may be made by its manufacturer, is not guaranteed or endorsed by the publisher.
